# Supposed to Have Been Lost at Sea

**Published:** 1853-10

**Authors:** 


*Supposed to have been Lost at Sea.*—We learn, with the most sincere regret, from a letter addressed to professor P. H. Austen, by Dr. J. F. Sanborn, of Beverly, Mass., that Dr. D. G. Varney, who went to California in 1849, sailed from thence for the Sandwich Islands in a small vessel, which not having since been heard from, is supposed to have foundered at sea on her passage. Dr. Varney graduated with high honors, at the commencement of the Baltimore College of Dental Surgery, in 1848, and some of the prettiest specimens of mechanical dentistry in the Museum of this institution, were presented by him to the Faculty on the occasion of his examination. He was a young gentleman of most persevering industry, good moral character, gentlemanly deportment, and of high promise. We hope, however, that the melancholy fate apprehended by his friends, may not have befallen him.

